# Hippocampal Interneurons are Required for Trace Eyeblink Conditioning in Mice

**DOI:** 10.1007/s12264-021-00700-0

**Published:** 2021-05-15

**Authors:** Wei-Wei Zhang, Rong-Rong Li, Jie Zhang, Jie Yan, Qian-Hui Zhang, Zhi-An Hu, Bo Hu, Zhong-Xiang Yao, Hao Chen

**Affiliations:** 1 Department of Physiology, College of Basic Medical Sciences, Army Medical University, Chongqing, 400038 China; 2 Department of Foreign Language, College of Basic Medical Sciences, Army Medical University, Chongqing, 400038 China; 3 Brain and Intelligence Research Key Laboratory of Chongqing Education Commission, Army Medical University, Chongqing, 400038 China; 4 Experimental Center of Basic Medicine, College of Basic Medical Sciences, Army Medical University, Chongqing, 400038 China

**Keywords:** Hippocampus, Interneuron, Trace eyeblink conditioning, Sustained activity, Associative learning

## Abstract

While the hippocampus has been implicated in supporting the association among time-separated events, the underlying cellular mechanisms have not been fully clarified. Here, we combined *in vivo* multi-channel recording and optogenetics to investigate the activity of hippocampal interneurons in freely-moving mice performing a trace eyeblink conditioning (tEBC) task. We found that the hippocampal interneurons exhibited conditioned stimulus (CS)-evoked sustained activity, which predicted the performance of conditioned eyeblink responses (CRs) in the early acquisition of the tEBC. Consistent with this, greater proportions of hippocampal pyramidal cells showed CS-evoked decreased activity in the early acquisition of the tEBC. Moreover, optogenetic suppression of the sustained activity in hippocampal interneurons severely impaired acquisition of the tEBC. In contrast, suppression of the sustained activity of hippocampal interneurons had no effect on the performance of well-learned CRs. Our findings highlight the role of hippocampal interneurons in the tEBC, and point to a potential cellular mechanism subserving associative learning.

**Supplementary Information:**

The online version contains supplementary material available at 10.1007/s12264-021-00700-0.

## Introduction

Learning to associate two events that occur separately in time is critical for animals to produce behavioral responses with appropriate timing and strength [1, 2]. Research has identified the neural mechanisms underlying associative learning [3, 4], but how this time-linked memory is acquired is not yet fully understood.

Evidence has accumulated that the hippocampus is one of the brain areas critically involved in associative learning. For instance, lesions of the dorsal hippocampus before training prevents subsequent associative learning [5–9]. Likewise, blockade of NMDA receptors in the dorsal hippocampus severely impairs the learning of associations between time-separated stimuli [10, 11]. Consistent with these lesion and inactivation studies, *in vivo* electrophysiological recordings show that a group of hippocampal pyramidal cells exhibits elevated activity and encodes information about discontiguous sets of stimuli [12]. Therefore, it has been hypothesized that the hippocampus functions to form associations between time-separated events, thereby supporting associative learning [2, 13].

Nevertheless, the contribution of distinct neuronal subtypes to associative learning remains unknown. The hippocampus contains a large number of GABAergic interneurons [14–16]. They play key roles in modulating the integration of synaptic inputs [17–19] and shaping the outputs of pyramidal cells [20], thus giving rise to oscillatory activity at different frequency bands [21–24]. These forms of network oscillatory activity are strongly correlated with associative learning performance [25, 26]. The above findings support the idea that the hippocampal interneurons participate in associative learning. However, there is still a lack of direct evidence illustrating whether and how the hippocampal interneurons are involved in associative learning.

To address this issue, we combined multiple-unit recordings and optogenetic techniques to measure and manipulate the firing activity of hippocampal interneurons while the freely-moving mice performed a hippocampus-dependent trace eyeblink conditioning (tEBC) task [7]. This type of conditioning is an ideal model in which to study the neural mechanisms of associative learning, because of its convenience in precisely controlling the delivery of a conditioned stimulus (CS; e.g., a light flash or a pure tone) and a reinforcing unconditioned stimulus (US; e.g., a corneal air-puff or a cutaneous electrical shock to the eyelid) [13]. Our results revealed the dynamic changes of CS-evoked sustained activity in hippocampal interneurons across distinct stages of tEBC acquisition. Moreover, we establish the causal relationship between the sustained activity of hippocampal interneurons and tEBC acquisition, verifying a pivotal role of hippocampal interneurons in this process. These findings thus extend the current knowledge of the cellular mechanisms underlying hippocampal involvement in associative learning.

## Materials and Methods

### Subjects

All experimental procedures were approved by the Animal Care Committee of Army Medical University. Wild-type C57/BL6 mice (*n* = 23, 3–6 months old, 20–25 g, both genders) were used as the subjects. In addition, optogenetic experiments were performed in GAD2-Cre mice (Stock no. 028867, Jackson Laboratory, USA, *n* = 20, 3–6 months old, 20–25 g, both genders) injected with either AAV2-CAG-FLEX-ArchT-GFP or AAV2-EF1α-DIO-GFP. Before the experiments and between the recording sessions, the mice were individually housed under a 12-h light-dark cycle with free access to food and water. All experiments were conducted between 8:00 and 20:00.

### Multiple Units Recording

The mice were anesthetized with isoflurane (0.6%–1.0% by volume in O_2_). The tungsten tetrodes (bare diameter, 20 μm; insulated diameter, 25 μm; California Fine Wire, USA) were implanted targeting the CA1 area of the left dorsal hippocampus (AP: − 1.9 mm, ML: − 1.65 mm, DV: 0.7 mm−0.8 mm from bregma). During 5–7 days of postoperative recovery, the tetrodes were moved (approximately 70 μm/day) until they reached the pyramidal cell layer characterized by a large-amplitude sharp wave-ripple (SWR). *In vivo* electrophysiological signals were continuously recorded at 20 kHz using a RHD2000 interface board (Intan Technology, C3334) and stored for offline analysis [27]. The local field potential in the dorsal hippocampus was sampled at 1250 Hz. All of the electrophysiological data were visualized using NeuroScope software (http://neurosuite.sourceforge.net) [28]. After each experiment, the brain was extracted for histological analysis.

### Behavioral Procedure

Following postoperative recovery, the mice were adapted to a containing box (45 cm × 25 cm × 20 cm) and the preamplifier plug-in and plug-out procedures, ensuring they behaved naturally during training and recording. The habituated mice were given daily behavioral training. For trace eyeblink conditioning (tEBC) training, the CSs were LED pulses of blue light flashes (150-ms in duration), while the USs were 100-ms air-puffs directed to the left cornea. The CS offset was separated from the US onset by a 250-ms trace interval. Daily tEBC training consisted of 100 CS–US paired presentation trials. The inter-trial interval (ITI) varied from 18 to 28 s with a mean value of 23 s. Pseudo-conditioned mice received 100 CSs and 100 USs per training day (mean ITI = 11.5 s), but the two stimuli were explicitly unpaired. All mice were trained for 4 consecutive days.

### Virus Injection and Diode-tetrode Assembly Implantation

Either AAV2-CAG-FLEX-ArchT-GFP or AAV2-EF1α-DIO-GFP (OBiO, China) was injected bilaterally into the hippocampus of GAD2-Cre mice (AP: − 1.9 mm, ML: ± 1.25, 1.50, and 1.75 mm, DV: 1.10 mm; 150 nL per site) using a microinjector (Nanoject II, Drummond Scientific). After the injection, the craniotomy was covered by low-viscosity silicone (Kwik-Cast^TM^, WPI) and the skin was sutured. After 4 weeks of post-injection viral expression, diode-tetrode assemblies were implanted bilaterally into the dorsal hippocampus of GAD2-cre mice (AP: − 1.9 mm, ML: ± 1.6 mm, DV: 1.0 mm). Fabrication of the diode-tetrode assembly has been previously described in detail [29]. Briefly, a 200-μm optic fiber (0.37 NA, FT200EMT, Thorlabs, Germany) was combined with the tetrode array. The light power was measured with an optical power meter (PM100D, Thorlabs). After the surgery, the mice were individually housed and allowed to recover for 5–7 days.

### Optogenetic Manipulation

During tEBC training, laser stimulation was delivered through the optical fibers. Laser diodes were activated by a driver (LDC–205C, Thorlabs) under the control of a pulse generator (Pulse Pal, Sanworks) [30]. The green laser (520 nm in wavelength; 15 mW/mm^2^–25 mW/mm^2^ at the fiber tip) was triggered by the CS and lasted for 400-ms in each trial to cover the periods of both the CS and trace interval.

### Data Analysis

#### EMG Analysis

The electromyography (EMG) signal from the left upper orbicularis oculi muscle was used to detect eyelid movement [31]. The eyeblink response was defined based on an algorithm as follows: (1) EMG data were collected, rectified and integrated with a time constant of 1-ms; (2) 300-ms EMG amplitude values before the CS onset were averaged cross 100 trials in each session to quantify the daily baseline and standard deviation (SD); (3) an invalid trial was identified when the 300-ms EMG amplitude values were at least 4 SDs above the daily baseline, otherwise a valid trial was identified; (4) significant eyeblink responses were detected in the valid trails in which the EMG amplitude exceeded the baseline by 4 SDs for at least 25-ms; and (5) an eyeblink response detected during 51-ms–400-ms after the CS onset was counted as a conditioned eyeblink response (CR).

#### Spike Sorting

The spikes were sorted using the procedures introduced in our recent report [32]. In brief, the spikes were extracted from high-pass filtered signals off-line, and their waveforms projected onto a common basis obtained by principal component analysis of the filtered data. Single-unit spikes were isolated off-line using both semi-automatic clustering with KlustaKwik software (http://klustakwik.sourceforge.net/) and manual clustering with Klusters software (http://neurosuite.sourceforge.net/). The accuracy of unit clustering was further verified by confirming the presence of a 2-ms refractory period devoid of spikes in the autocorrelogram of a putative single unit [28].

#### Unit Classification

Single units were classified into either putative pyramidal cells (Pyrs) or interneurons (INs) based on their firing rate and waveform (i.e., the trough-to-peak duration and spike width). Units with an average firing rate <6 Hz and a peak-to-trough duration >0.35-ms were classified as putative Pyrs, while units with an average firing rate ≥6 Hz and a peak-to-trough duration ≤0.35-ms were classified as putative INs [29, 33]. The spikes recorded during each valid trial were assigned to 50-ms time bins, beginning 1000-ms before and extending 1000-ms after the CS onset. The firing rate (FR) for each bin across all valid trials was calculated. The mean FR for the 20 bins before the CS onset was used as the baseline, and their SDs were computed. The firing rate of each bin was normalized by using the Z score as follows (#bin refers to an arbitrary time bin):

$$ \text{Z} = {{\left( {FR_{{\# {\text{bin}}}} - {\text{mean}}\;FR_{{{\text{baseline}}}} } \right)} \mathord{\left/ {\vphantom {{\left( {FR_{{\# {\text{bin}}}} - {\text{mean}}\;FR_{{{\text{baseline}}}} } \right)} {{\text{SD }}\left( {FR_{{{\text{baseline}}}} } \right)}}} \right. \kern-\nulldelimiterspace} {{\text{SD }}\left( {FR_{{{\text{baseline}}}} } \right)}}. $$

### Histology

Electrolytic lesions (30-μA for 10 s, DC current) were made following the electrophysiology experiments to confirm the recording site of the tetrode assembly. 48 h later, the mice were transcardially perfused with 4% paraformaldehyde (PFA; prepared in 0.1 mol/L phosphate buffer, pH 7.4). After post-fixation with 4% PFA for 8 h followed by dehydration with 30% sucrose PBS for 48 h, each brain was cut into 50-μm coronal sections (CM1900, Leica) and immune-stained with DAPI to visualize nuclei.

GAD2-Cre mice were transcardially perfused immediately following the optogenetic experiments to confirm the viral expression and optical fiber position. 50-μm coronal sections were cut using the above procedure. The sections were prepared for 1-h permeabilization with 0.5% TritonX-100 in PBS followed by 1 h blocking with 5% BSA. Afterwards, the sections were incubated overnight with rabbit antibody to GABA (1:800, A2052, Stock no. A2052, Sigma, USA) at 4 °C followed by 2 h incubation with Alexa Fluor 568-conjugated donkey antibody to rabbit (1:800, Stock no. A10042, Thermo Scientific, USA) at room temperature. The sections were incubated with DAPI and mounted in Fluoromount (stock no. 0100-01, Southern Biotech). All sections were imaged using a fluorescence microscope (BX53, Olympus, Japan).

### Statistics

Data are expressed as the mean ± SEM unless otherwise noted. We first tested the normality of each dataset using Shapiro-Wilk test. Parametric tests were used if the dataset passed the normality test. Otherwise, the Wilcoxon rank sum test was used. The statistical significance for behavioral analysis was determined by one-way or two-way ANOVAs with repeated measures. Difference in firing activity between two groups (paired *vs* unpaired) was determined by the independent *t* test (2-tailed). Paired *t* tests were used to determine the significance of differences in firing activity between two states (CR *vs* no-CR) and the effect of optogenetic manipulation on the performance of well-learned CRs. A value of *P* <0.05 was considered to be significant for all tests. Significance levels of data are denoted as *P <0.05, **P <0.01, and ***P <0.001. *P* >0.05 was considered to be not-significant and is denoted as *n.s*.

## Results

### Behavioral Performance

**Fig. 1:**
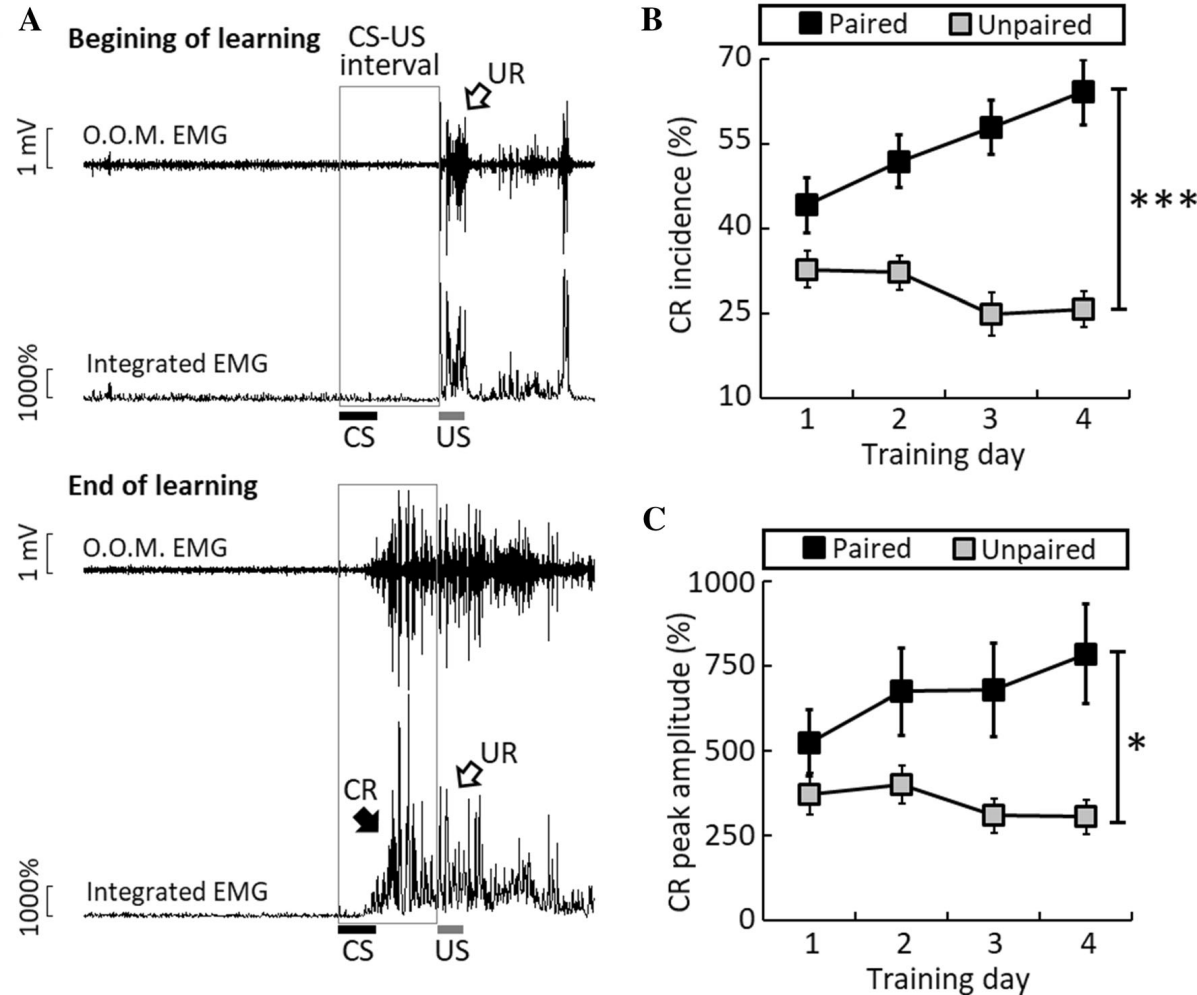
Acquisition of trace eyeblink conditioning in freely-moving mice. **A** Example of eyelid responses from a mouse before (upper) and after learning (lower). The CS was a 150-ms LED light, while the US was a 100-ms air-puff to the cornea. The interval between the CS offset and the US onset was 250-ms. The top trace of each panel shows the raw orbicularis oculi muscle (O.O.M.) EMG, while the bottom trace shows the integrated EMG. **B**, **C** CR incidence (**B**) and CR peak amplitude **(C)** from the paired (*n* = 12, black squares) and unpaired (*n* = 11, grey squares) trained mice across 4 training days. Data are expressed as the mean ± SEM (*P <0.05, ***P <0.001, two-way ANOVAs with repeated measures).

To examine tEBC task-related interneuron activity in the dorsal hippocampus, we trained the freely-moving mice to acquire tEBC, in which a 250-ms trace interval was inserted between the light CS and the air-puff US (Fig. 1A). We found that the mice receiving CS-US paired presentations learned this task and exhibited adaptive CRs to the light CSs (Fig. 1A). This was further supported by the statistical results that the mice with CS-US paired training showed significant increases in the CR incidence and peak amplitude across 4 training days (paired group, *n* = 12 mice, CR incidence: *F*_3, 33_ = 7.678, *P* = 0.001; CR peak amplitude: *F*_3, 33_ = 3.785, *P* = 0.019, one-way ANOVAs with repeated measures, Fig. 1B, C). On average, the CR incidence increased from 44.1% ± 4.9% on day1 to 64.1% ± 5.8% on day 4. In contrast, the mice with unpaired training showed no sign of learning, as evidenced by the decreased CR levels across 4 training days (unpaired group, *n* = 11 mice, CR incidence: *F*_3, 30_ = 4.893, *P* = 0.007; peak amplitude: *F*_3, 30_ = 2.831, *P* = 0.055, one-way ANOVAs with repeated measures). Moreover, our statistical analysis revealed that the mice with paired training exhibited higher CR levels than those with unpaired training (main effects, CR incidence: *F*_1, 21_ = 26.794, *P* <0.001; CR peak amplitude: *F*_1, 21_ = 6.367, *P* = 0.020; Interaction, CR incidence: *F*_3, 63_ = 2.057, *P* = 0.115; CR peak amplitude: *F*_3, 63_ = 1.954, *P* = 0.222, two-way ANOVAs with repeated measures, Fig. 1B, C). These results indicated that tEBC was acquired in the CS-US mice with paired training across 4 training days. It should be noted that the higher CR level was unlikely to result from better learning in the mice with paired training because they showed CR scores similar to those of the mice with unpaired training at the beginning of training on day 1 (Fig. S1).

### *In vivo* Electrophysiological Identification of Putative Interneurons

**Fig. 2:**
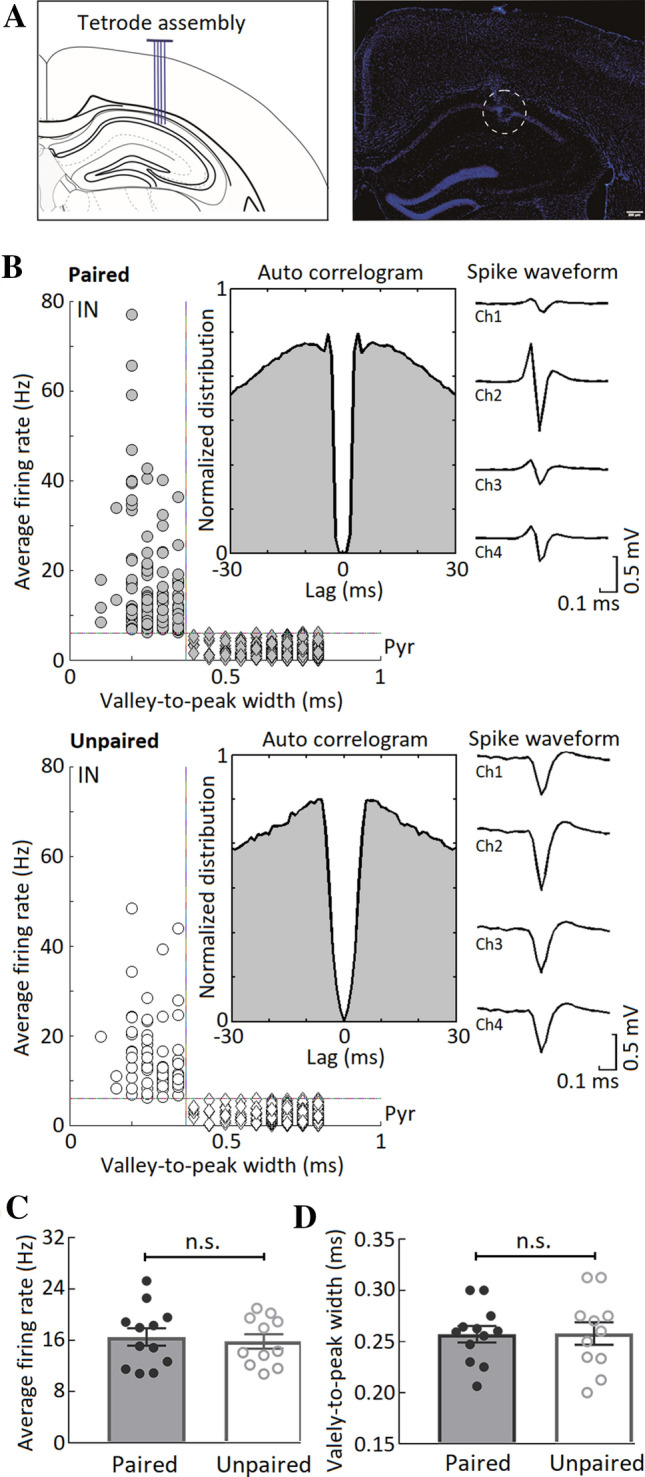
Classification of hippocampal units into putative interneurons and pyramidal cells. **A** Location of recording sites in the dorsal hippocampus. Left, schematic of tetrode recording in the dorsal hippocampus; right: a DAPI-stained coronal section illustrating a representative recording site in the dorsal hippocampus (dashed circle; scale bar, 200-μm). **B** Scatter plots of valley-to-peak width of spike waveforms *vs* average firing rates for 1101 and 788 hippocampal units isolated from mice with paired (upper, *n* = 12 mice, across 4 sessions) and unpaired training (lower, *n* = 11 mice, across 4 sessions). Representative spike train autocorrelations and spike waveforms of the representative INs from mice with paired and unpaired training. **C** There are no significant differences in the average firing rates of putative INs between the paired (*n* = 104, across 12 mice in 4 sessions) and unpaired trained mice (*n* = 63, across 11 mice in 4 sessions). **D** There are no significant differences in the valley-to-peak widths of putative INs between the paired (*n* = 104, across 12 mice in 4 sessions) and unpaired trained mice (*n* = 63, across 11 mice in 4 sessions). Data are expressed as the mean ± SEM (n.s., not significant, two-tailed independent *t* test).

We identified the recorded hippocampal units as either putative interneurons (INs) or putative pyramidal cells (Pyrs) by discharge rates and the spike waveform. Recording in the dorsal hippocampus was determined by both *post hoc* histological examination (Fig. 2A) and the large amplitude of sharp wave-ripples (Fig. S2). Hippocampal INs have been reported to exhibit high discharge rates (>6 Hz) and narrow spike width (valley-to-peak width ≤0.35-ms) [29, 33]. By using these criteria, 104 putative INs were identified in the mice with CS-US paired presentation (across 12 mice, average 8.7 units per mouse, Fig. 2B), and 63 putative INs were identified in the mice with unpaired training (across 11 mice, average 5.7 units per mouse, Fig. 2B). There were no significant differences in either average firing rate or spike width of putative INs between the mice with paired and unpaired training (average firing rate, paired group: 16.5 ± 1.4 Hz, unpaired group: 15.8 ± 1.2 Hz, *t*_21_ = 0.397, *P* = 0.695, Fig. 2C; spike width, paired group: 0.257 ± 0.008-ms, unpaired group: 0.258 ± 0.012 ms, *t*_21_ = − 0.051, *P* = 0.960, independent *t* tests, Fig. 2D). It should be noted that, in the current study, 997 putative Pyrs were identified in the mice with CS-US paired training (across 12 mice, average 83.1 units per mouse, Fig. 2B), and 725 putative Pyrs were identified in the unpaired mice (across 11 mice, average 65.9 units per mouse, Fig. 2B).

### Hippocampal Interneurons Show Sustained Activity During tEBC

**Fig. 3:**
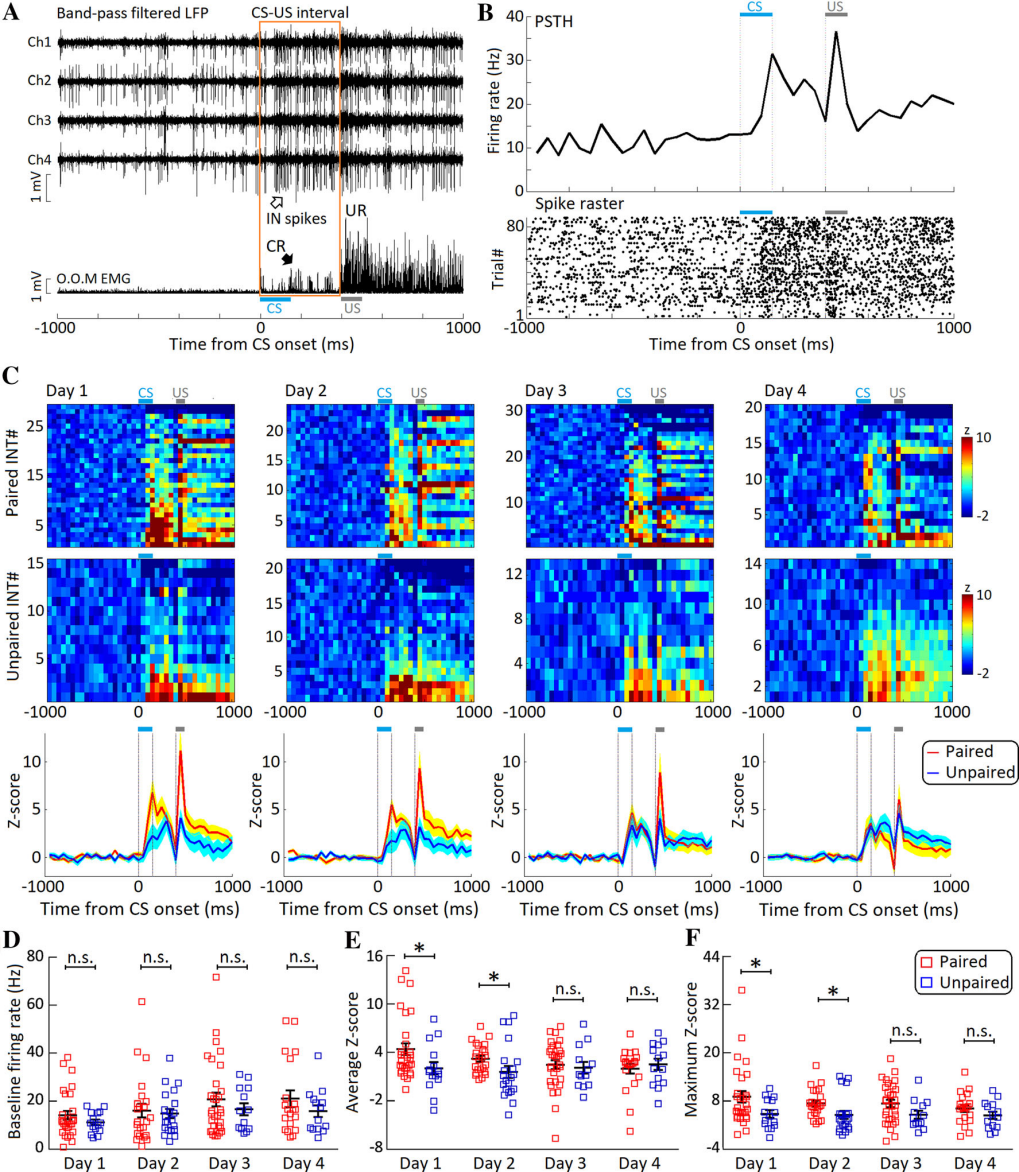
CS-evoked sustained activity of hippocampal interneurons (INs) during tEBC. **A** Upper, tetrode recording of CS-evoked sustained activity in a representative IN during tEBC training; lower, orbicularis oculi muscle (O.O.M.) EMG trace peristimulus. **B** Peristimulus time histogram (PSTH, upper) and raster plot (lower) of the spiking responses of a representative IN to light CSs. **C** CS-evoked sustained activity of INs in mice with paired and unpaired training across 4 training days. Upper, rows, heatmaps of Z-score-transformed average PSTHs for individual INs; columns, time bins relative to CS onset (50-ms bin-width); lower, plots of the average Z score responses for INs in mice with paired (red) and unpaired (blue) training across 4 training days. **D** Average baseline firing rates of INs in mice with paired (red squares) and unpaired (blue squares) training. **E, F** CS-evoked sustained activity of INs in mice with paired training (red squares) was significantly stronger than that in mice with unpaired training (blue squares) on days 1 and 2. In contrast, CS-evoked sustained activity in INs in mice with paired training (red squares) were comparable to those in mice with unpaired training (blue squares) on days 3 and 4. Data are expressed as the mean ± SEM (*P <0.05, n.s., not significant; two-tailed independent *t* test).

We examined the characteristics of hippocampal IN activity during tEBC training. Strong CS-evoked firing activity was recorded in the INs (Fig. 3A), and it did not cease until the trace interval period (Fig. 3B). On the first 2 days, there were no significant differences in the baseline activity of INs between the mice with paired and unpaired training (day 1: *t*_42_ = 1.137, *P* = 0.262; day 2: *t*_43_ = 0.349, *P* = 0.729; independent *t* tests, Fig. 3D). However, the CS-evoked sustained activity of INs in mice with paired-training was greater than in those with unpaired training (average *Z* score, day 1: *t*_42_ = 2.070, *P* = 0.045; day 2: *t*_43_ = 2.128, *P* = 0.039; maximum *Z* score, day 1: *t*_42_ = 2.039, *P* = 0.048; day 2: *t*_43_ = 2.364, *P* = 0.023; independent *t* tests, Fig. 3C–E). Moreover, on the first 2 days, we found a greater proportion of sustained active INs in the paired-training mice than in the unpaired-training mice (paired group: 96.2% (51 of 53 units) *vs* unpaired group: 61.1% (22 of 36 units), *χ*^*2*^ = 17.927, *df* = 1, *P* <0.001, Pearson χ^*2*^ test, Fig. 3C). These results suggest that hippocampal INs manifest strong CS-evoked sustained activity during the early acquisition of tEBC.

On days 3 and 4, however, the CS-evoked sustained activity of hippocampal interneurons in paired-training mice tended to diminish (Fig. 3C). This activity on day 4 was lower than on day 1 (average Z score, day 1 *vs* day 4: *t*_47_ = 2.408, *P* = 0.020; maximum Z score, day1 *vs* day 4: *t*_47_ = 1.567, *P* = 0.124; independent *t* tests, Fig. 3C–E). Moreover, no significant differences in the CS-evoked sustained activity of INs were found between the paired and unpaired training mice (average Z score, day 3: *t*_42_ = 0.384, *P* = 0.703; day 4: *t*_32_ = − 0.547, *P* = 0.194; maximum Z score, day 3: *t*_42_ = 1.872, *P* = 0.075; day 4: *t*_32_ = 1.326, *P* = 0.194; independent *t* tests, Fig. 3C–E). Likewise, comparable proportions of sustained active INs were found between the paired and unpaired training mice (paired group: 82.4% (42 of 51 units) *vs* unpaired group: 66.7% (18 of 27 units), *χ*^*2*^= 2.447, *df* = 1, *P* = 0.118, Pearson *χ*^*2*^ test, Fig. 3C). These results suggest that stronger CS-evoked sustained activity of hippocampal INs only occurs during the early, rather than the late acquisition of tEBC.

**Fig. 4:**
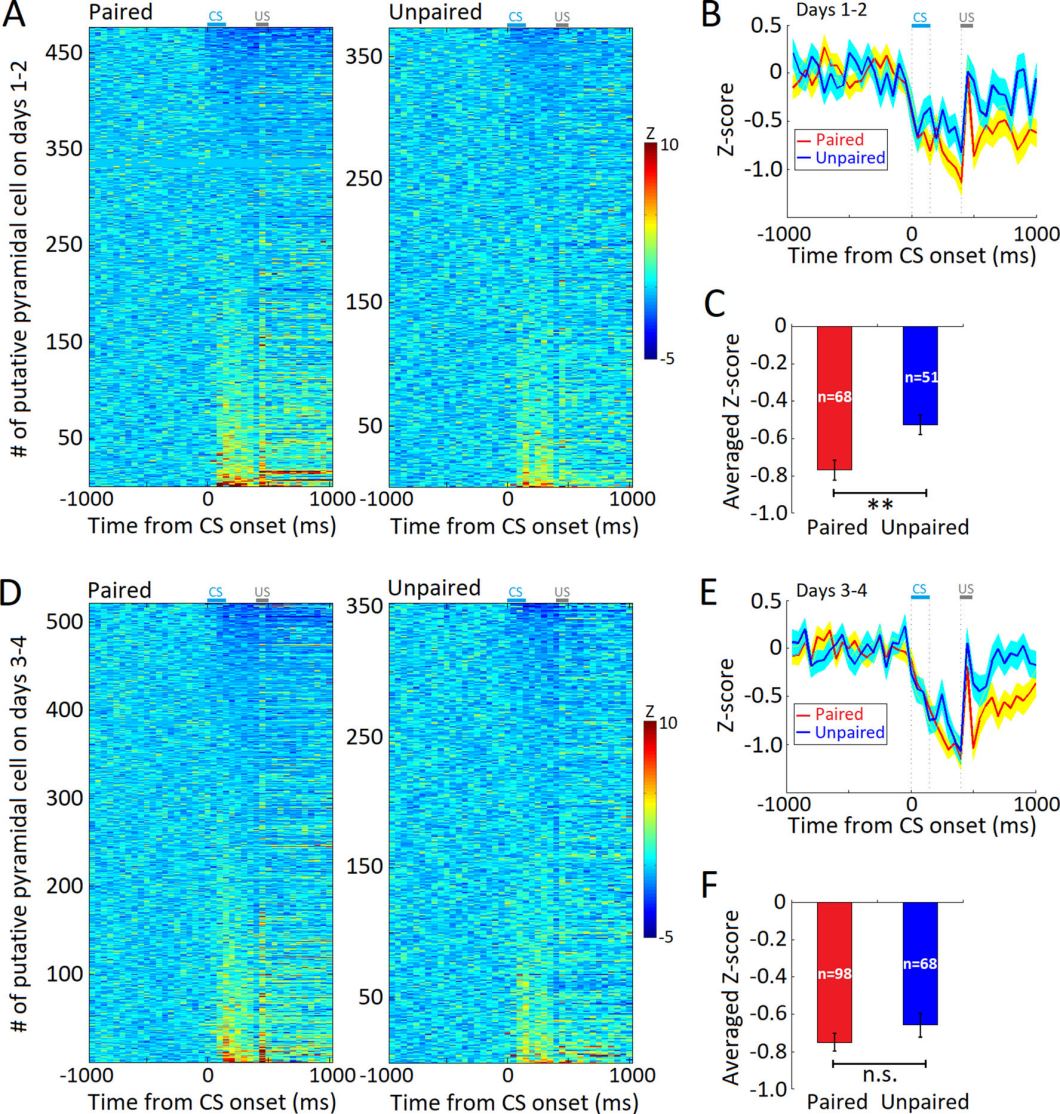
CS-evoked firing activity of putative pyramidal cells (Pyrs) during tEBC training. **A** Pseudo-colored, baseline normalized peri-CS histograms of all classified Pyrs during the early acquisition of tEBC in mice with paired (left, *n* = 476) and unpaired training (right, *n* = 373). Lighter shades represent increases in firing activity. Cells are sorted according to the magnitude of the change in CS-evoked firing activity. **B** Average peri-event responses of CS-evoked decreased firing in Pyrs in the early-learning stage. **C** CS-evoked decrement in firing of Pyrs in mice with paired training is greater than that in mice with unpaired training (days 1–2; paired, *n* = 68 units; unpaired, *n* = 51 units, *t*_117_ = − 3.197, *P* = 0.002, independent *t* test). **D** Pseudo colored, baseline normalized peri-CS histograms of all classified Pyrs during the late acquisition of tEBC in the mice with paired (left, *n* = 521) and unpaired training (right, *n* = 352). Lighter shades represent increases in firing activity. Cells are sorted according to the magnitude of the change in CS-evoked firing activity. **E** Average peri-event responses of Pyrs with CS-evoked decreased firing in the late-learning stage. **F** The CS-evoked decrement in firing of Pyrs in mice with paired training is comparable to that in those with unpaired training (days 3–4; paired, *n* = 98 units, unpaired, *n* = 68 units, *t*_164_ = − 1.224, *P* = 0.223, independent *t* test). Data are expressed as the mean ± SEM (**P <0.01, n.s., not significant).

In parallel with greater sustained activity of hippocampal INs during the early acquisition of tEBC, we also recorded decreased CS-evoked firing in putative hippocampal Pyrs. Putative Pyrs in the mice with paired training showed decreased firing activity at 138.0 ± 6.7-ms after the CS onset, and showed a greater decrement in firing than the mice with unpaired training (days 1–2, paired group: *n* = 68 units, unpaired group: *n* = 51 units, *t*_117_ = − 3.197, *P* = 0.002, independent *t* test, Fig. 4A–C). Again, such a difference in firing activity did not occur in the late acquisition of tEBC (days 3–4, paired group: *n* = 98 units, unpaired group: *n* = 68 units, *t*_164_ = − 1.224, *P* = 0.223, independent *t* test, Fig. 4D–F). These results provide further evidence for the stronger inhibition of hippocampal network activity during the early, rather than the late acquisition of tEBC.

### Sustained Activity of Hippocampal Interneurons Bias between CR and no-CR States

**Fig. 5:**
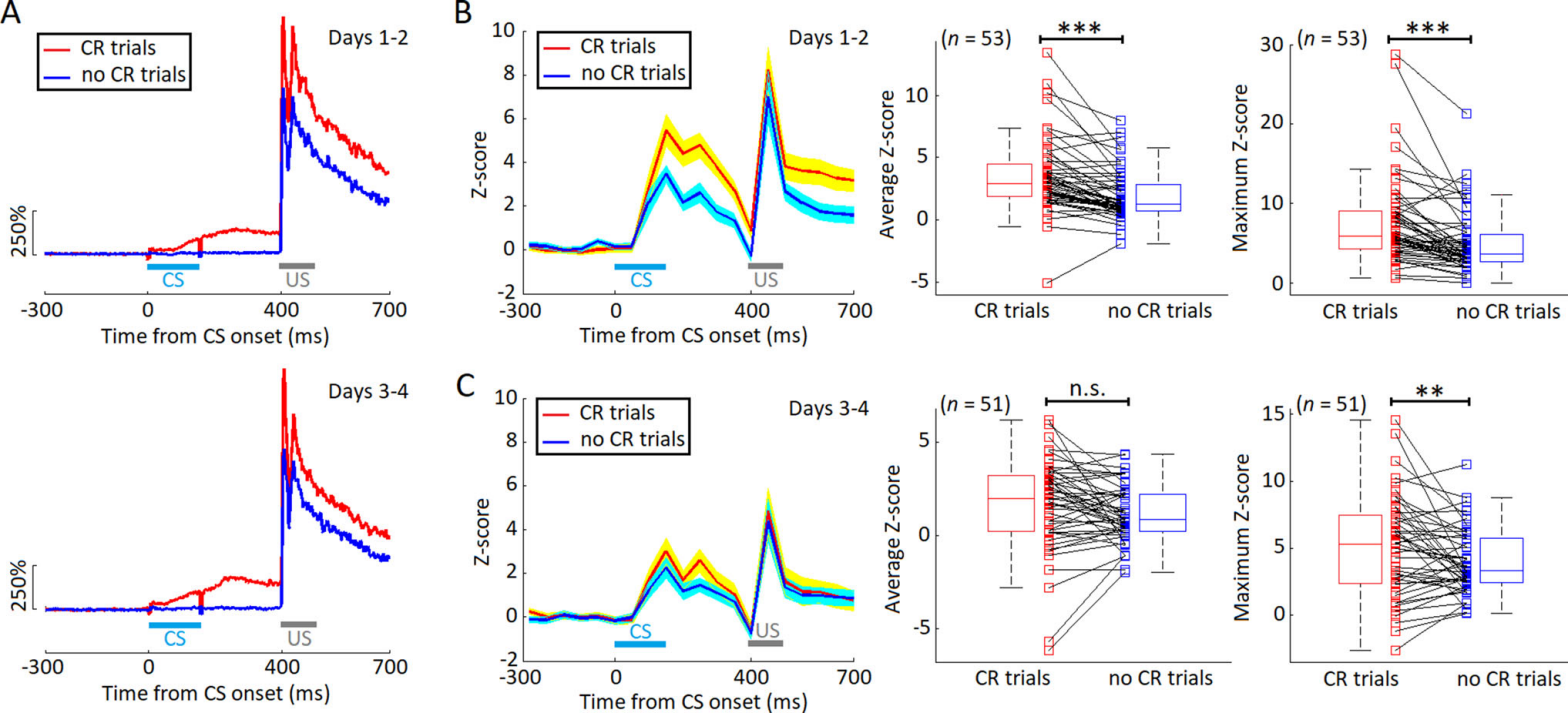
Hippocampal interneuron (IN) activity in CR *vs* no-CR trials. **A** Average CR (red) and no-CR trials (blue) across mice with CS-US paired training (*n* = 12) during the early (days 1–2, upper) and late (days 3–4, lower) stages of tEBC acquisition. **B** On days 1 and 2, the sustained activity of INs in the CR trials (red) is significantly stronger than that in the no-CR trials (blue). **C** On days 3 and 4, the sustained activity of INs in the CR trials (red) is comparable to those in the no-CR trials (blue) (***P <0.001, **P <0.01, n.s., not significant, paired *t* test.

In the mice with paired training, the hippocampal INs increased their firing activity at 131.0 ± 7.5 ms after the CS onset (Fig. 5A, B), and this was in parallel with the occurrence of the CR. Therefore, we hypothesized that these INs contributed to the CR acquisition. To test this hypothesis, we first compared the sustained activity of the INs between the CR and no-CR states. This showed that, on days 1 and 2, sustained activity of INs in the CR trials was significantly higher than that in the no-CR trials (*n* = 53, average Z score: *t*_52_ = 5.368, *P* <0.001; maximum Z score: *t*_52_ = 5.660, *P* <0.001, paired *t* test, Fig. 5B), indicating that sustained IN activity was biased between the CR and no-CR states. In contrast, no significant bias was found in the sustained activity of INs between the CR and no-CR states on days 3 and 4 (*n* = 51, average Z score: *t*_50_ = 1.772, *P* = 0.083; maximum Z score: *t*_50_ = 2.695, *P* = 0.0097, paired *t* test, Fig. 5C). Together, these results suggest that the sustained activity of hippocampal INs is strongly correlated with the CR occurrence during the early stage of tEBC acquisition.

### Optogenetic Suppression of Hippocampal Interneurons Impairs the Acquisition of tEBC

**Fig. 6:**
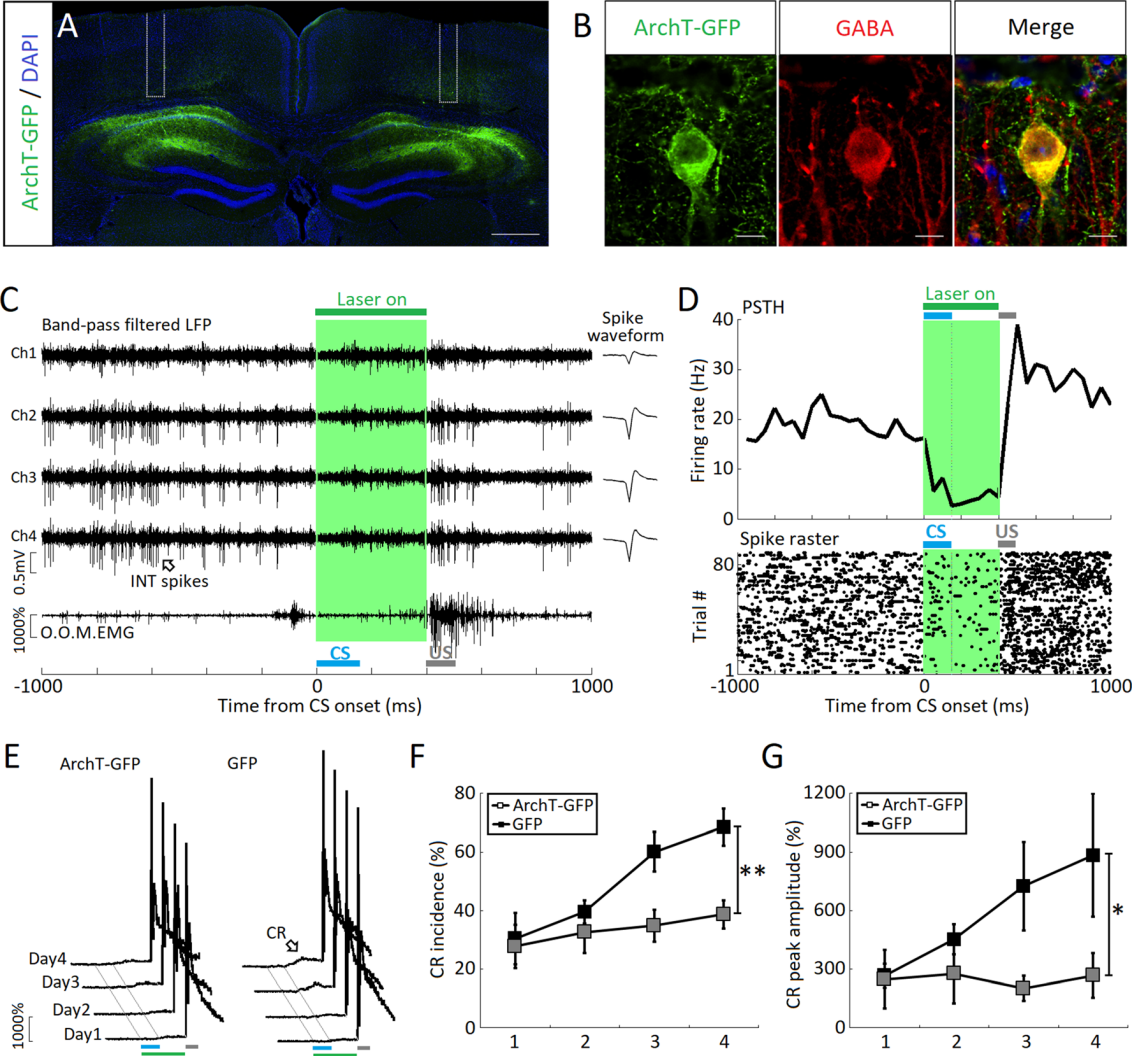
Optogenetic suppression of sustained activity in hippocampal interneurons (INs) impairs the acquisition of tEBC. **A** Coronal section of a GAD2-Cre mouse brain showing ArchT-GFP expression (green) stained with DAPI (blue) in the dorsal hippocampus. **B** Magnified images showing overlap of ArchT-GFP (green) expression and GABA immunoreactivity (red) in the hippocampal CA1 area of a GAD2-cre mouse (scale bars, 50-μm). **C** Upper, tetrode recording showing the optogenetic suppression of sustained activity in a representative IN; lower, orbicularis oculi muscle (O.O.M.) EMG during peri-optogenetic stimulation. **D** PSTH (upper) and raster plot (lower) illustrating the spiking responses of the representative IN in **C** to 400-ms green light stimulation. **E** Average eyelid responses of valid trials across 4 training days for GAD2-Cre mice with viral injection of AAV-CAG-ArchT-GFP (*n* = 7, left) and GAD2-Cre mice with viral injection of AAV-DIO-GFP (*n* = 7, right). **F, G** CR incidence (**F**) and CR peak amplitude (**G**) measured in the ArchT-GFP (*n* = 7, filled squares) and GFP (*n* = 7, open squares) groups across 4 training days. Data are expressed as the mean ± SEM (**P <0.01, *P <0.05, two-way ANOVAs with repeated measures).

The sustained activity of hippocampal INs was correlated with behavioral outcome in terms of CR and no-CR states, indicating that this activity contributes to the acquisition of tEBC. Consequently, we further quantified this contribution by acutely suppressing the firing response. For this purpose, we tested another group of mice, in which the inhibitory opsin ArchT was virally expressed in hippocampal INs (Fig. 6A, B). We found that presentation of 400-ms green laser light after the CS onset suppressed the sustained activity of INs (Fig. 6C, D), and suppression of the INs disinhibited (increased) the firing activity of putative Pyrs (Fig. S3). Importantly, suppressing sustained IN activity resulted in a marked impairment of CR acquisition (main effects: CR incidence: *F*_1, 12_ = 14.143, *P* = 0.003; CR peak amplitude: *F*_1, 21_ = 4.789, *P* = 0.049; interaction: CR incidence: *F*_1, 12_ = 0.786, *P* = 0.393; CR magnitude: *F*_1, 12_ = 0.455, *P* = 0.513, two-way ANOVAs with repeated measures, Fig. 6E–G). These results suggest that sustained activity of hippocampal INs, rather than increased firing activity of Pyrs, is required for the acquisition of tEBC.

### Optogenetic Suppression of Hippocampal Interneurons Fails to Impact the Performance of Well-learned CRs

**Fig. 7:**
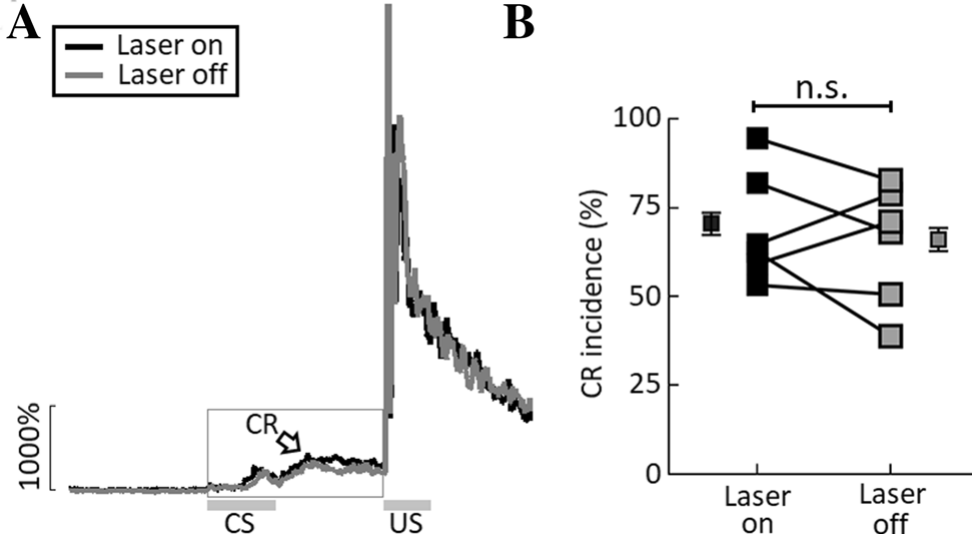
Optogenetic suppression of hippocampal interneurons (INs) fails to impair the performance of well-learned CRs. **A** Average eyelid movement traces illustrating the effect of optogenetic suppression of INs on the performance of well-learned CRs (*n* = 6 mice; arrow, CRs; open rectangle, delivery of green laser light in the CS–US period). **B** Optogenetic suppression of INs during the CS-US period has no effect on the CR incidence when the CRs are well-learned (*n* = 6 mice). Data are expressed as the mean ± SEM; n.s., not significant, paired *t* test).

If the sustained activity of hippocampal INs only participates in the early acquisition of tEBC, one would expect that acutely blocking this activity would have minimal effects on the performance of well-learned CRs. We therefore tested another group of GAD2-cre mice that had established a well-learned CR. Since the inhibitory opsin ArchT was virally expressed in dorsal hippocampal INs, we could optogenetically inhibit their sustained activity during the interval between the CS and the US. We found that inhibition of the sustained activity of the INs caused no significant change in the incidence of well-learned CRs (CR incidence: laser on: 67.4 ± 8.2% *vs* laser off: 67.1 ± 6.4%, *t*_5_ = − 0.047, *P* = 0.965, paired *t* test, *n* = 6 mice, Fig. 7), suggesting that the sustained activity of hippocampal INs is not required for the performance of well-learned CRs.

## Discussion

In this study, we combined multiple-unit recording and optogenetics to investigate the role of hippocampal INs in a hippocampus-dependent tEBC task. We found that, during the acquisition of tEBC, the INs exhibited CS-evoked firing activity sustained during the time interval between the CS and the US. This activity was correlated with CR performance in a learning stage-specific manner. Moreover, optogenetic suppression of the sustained activity in the INs severely impaired the acquisition of tEBC. In contrast, suppression of this sustained activity had no effect on the performance of well-learned CRs. Our results provide direct evidence that hippocampal INs participate in associative learning *via* CS-evoked sustained activity.

Although the hippocampus is thought to support the association among time-separated events [2, 34–36], the underlying cellular mechanisms are not fully understood. It has been suggested that transgenic mice with altered IN function display impaired associative learning in hippocampus-dependent tasks [37], and the excitability and activity of hippocampal INs are increased with associative learning [38, 39]. We report here associative learning-induced sustained activity in hippocampal INs. The CS-evoked sustained activity was recorded in 89.4% (93 of 104 units) hippocampal neurons during tEBC, indicating that it is a homogeneous feature of hippocampal INs in this process. Notably, optogenetic suppression of the sustained activity impaired the acquisition of tEBC. Therefore, our results not only provide direct evidence that hippocampal INs are critically involved in the acquisition of tEBC, but also suggest that their sustained activity is one of the cellular mechanisms required for successful associative learning.

The hippocampus is thought to play a time-limited role in the acquisition of tEBC [40–42]. Consistent with this, we found that greater sustained activity in hippocampal INs was correlated with CR occurrence in the early acquisition of tEBC, suggesting that CS-evoked sustained activity of these INs is possibly involved in the initial learning that requires short-term memory, for which CS-evoked activity is sustained up to the onset of US. At the late learning stage, however, the magnitude of this sustained activity began to decline. Therefore, it is reasonable to propose that the sustained activity of the INs no longer differs from that with unpaired training on days 3–4 because long-term memory is established. In support of this, suppression of the sustained activity in the INs had no evident effect on the performance of well-learned CRs. Our findings thus indicate that dynamic change in the sustained activity of hippocampal INs is a candidate cellular process reflecting the differential involvement of the hippocampus in various stages of tEBC acquisition.

But how does the sustained activity of hippocampal interneurons participate in tEBC acquisition? Many studies have demonstrated that tEBC is mediated by cerebellar learning in response to forebrain-driven mossy fiber inputs that persist beyond CS offset to overlap with the US [43–47]. Among the forebrain areas, the hippocampus has been implicated because of the CS-evoked theta synchronization between the hippocampus and the cerebellum during tEBC [48, 49]. Electrophysiological studies have demonstrated that, at the population level, the activity of hippocampal Pyrs increases during the CS–US period [12]. This Pyr activity has been hypothesized to support the associations between time-separated events [2, 13]. Similar to the previous electrophysiological reports [12], we recorded ~40.1% (400/997) of the recorded Pyrs exhibited CS-evoked increased firing activity. Meanwhile, ~16.6% (166/997) of the recorded Pyrs showed CS-evoked decreased firing. The latency of the CS-evoked decreased firing in Pyrs was similar to that of the sustained activity in INs, implying these two responses might be correlated. Indeed, it has been suggested that the inhibitory IN inputs to excitatory principal cells can increase signal-to-noise in the brain [50, 51]. We thus speculated that the sustained IN activity might result in augmenting the signal-to-noise of hippocampal Pyr encoding during the CS–US period, and helps to shape and propagate the hippocampal outputs involved in tEBC acquisition [52].

It should be noted that more hippocampal Pyrs showed increased firing than that showed a decreased firing (Fig. 4A). This result seemed to contradict the fact of overall increased IN activity in the hippocampus. Indeed, it has been demonstrated that lateral inhibition allows a first assembly of Pyrs to suppress the activity of another assembly of Pyrs through the excitation of inhibitory INs [53]. Therefore, one possible explanation for our current findings is that the increased firing of Pyrs interacts with the sustained IN firing *via* collaterals, and this interaction in turn contributes to the lateral inhibition of neighboring Pyrs in the hippocampus [52–54].

Establishing an association between the CS and the US is a prerequisite for the acquisition of tEBC [7, 9, 11]. In this study, we found that both INs and Pyrs showed increased firing activity after the US onset. Previous studies suggested that the US-evoked firing activity is important because the CS-evoked activity would sharply decrease if no USs were further presented [55]. In most INs and some Pyrs we recorded, the CS-evoked firing activity was sustained up to the US-evoked activity period. Consequently, it is reasonable to hypothesize that, at the cellular level, such a firing pattern is a candidate mechanism bridging the time gap between two stimuli to mediate associative learning [53].

The results reported here add to our understanding of the role of inhibitory INs in the hippocampus during associative learning. However, considering the diversity of GABAergic INs in the hippocampus [14–16], the question arises as to the contribution of different subsets of hippocampal INs to associative learning. Consequently, future experiments combing fiber photometry with optogenetics to precisely record and control the activity of different subsets of hippocampal INs [17, 56, 57], are necessary to determine which type of IN(s) is essential for successful associative learning. In addition, a feature of the sustained activity was elevated spiking during the interval between the CS and US that was required for sustained maintenance [58, 59]. Therefore, future experiments can be done to vary the time interval between the CS and the US to better uncover the feature of sustained hippocampal activity.

In conclusion, previous studies have focused on the activity of hippocampal Pyrs, providing progress in research on the brain areas critically involved in associative learning. However, the activity and function of hippocampal INs in this process were neglected. Our current findings provide a mechanistic understanding of the properties of hippocampal IN activity that supports successful associative learning. Nevertheless, further experiments are needed to unravel how the sustained activity of hippocampal INs sculpts the activity of the Pyr network, so as to shape the outputs of the hippocampus and support the communication between the hippocampus and extra-hippocampal areas.

## Supplementary Information

Below is the link to the electronic supplementary material.

Supplementary file1 (PDF 415 kb)
