# Supplementary material for: Hippocampal Interneurons are Required for Trace Eyeblink Conditioning in Mice
Source: Neurosci Bull. 2021 May 15;37(8):1147–59. doi: 10.1007/s12264-021-00700-0 (PMC8353031; doi:10.1007/s12264-021-00700-0)
Supplement: Supplementary file 1 — Supplementary file1 (PDF 415 kb) [file 12264_2021_700_MOESM1_ESM.pdf]

Supplementary Materials

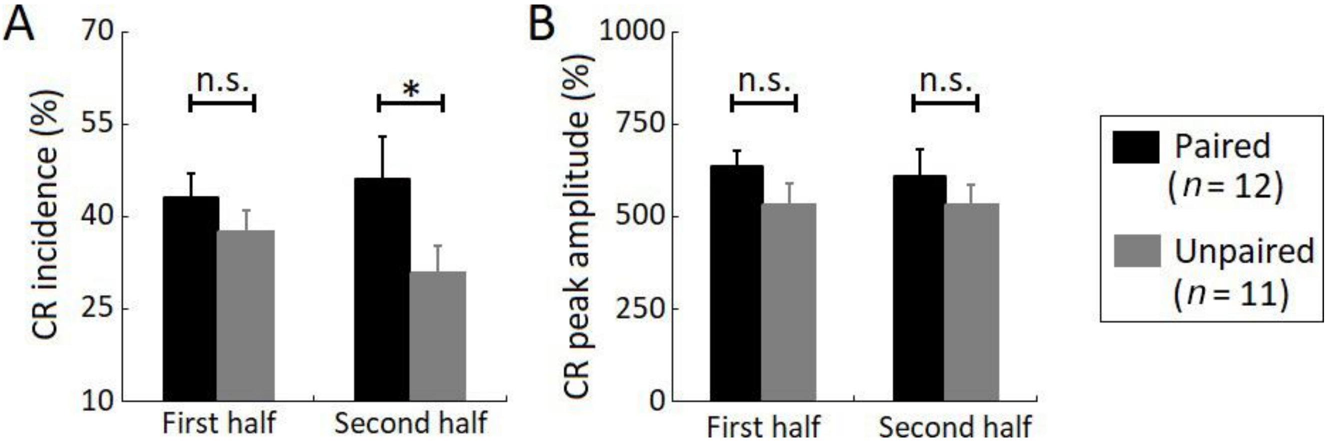

Fig. S1 CR acquisition on day 1. **A** CR incidence measured from the mice with paired (*n* = 12, black) and unpaired (*n* = 11, grey) training on day 1. There is no significant difference during the first half (trials 1–50) (paired group, 43.4% ± 3.8% *vs* unpaired group, 37.6% ± 3.6%, t_{21} = 1.169, *P* = 0.255, independent *t* test). By contrast, there is a significant difference during the second half (trials 51–100) (paired group, 46.4% ± 6.8% *vs* unpaired group, 30.9% ± 4.5%, t_{21} = 2.174, *P* = 0.045, independent *t* test). **B** CR peak amplitude measured from the mice with paired (*n* = 12, black) and unpaired (*n* = 11, grey) training. There is no significant difference between the paired and unpaired groups during either the first or the second half of training (First half: paired group, 638.9% ± 42.4% *vs*. unpaired group, 532.5% ± 60.9%, t_{21} = 1.579, *P* = 0.130; Second half: paired, 612.3% ± 71.3% *vs* unpaired, 535.9% ± 52.7%, t_{21} = 0.902, *P* = 0.377, independent *t* tests). Data are expressed as the mean ± SEM (**P* <0.05, n.s., not significant).

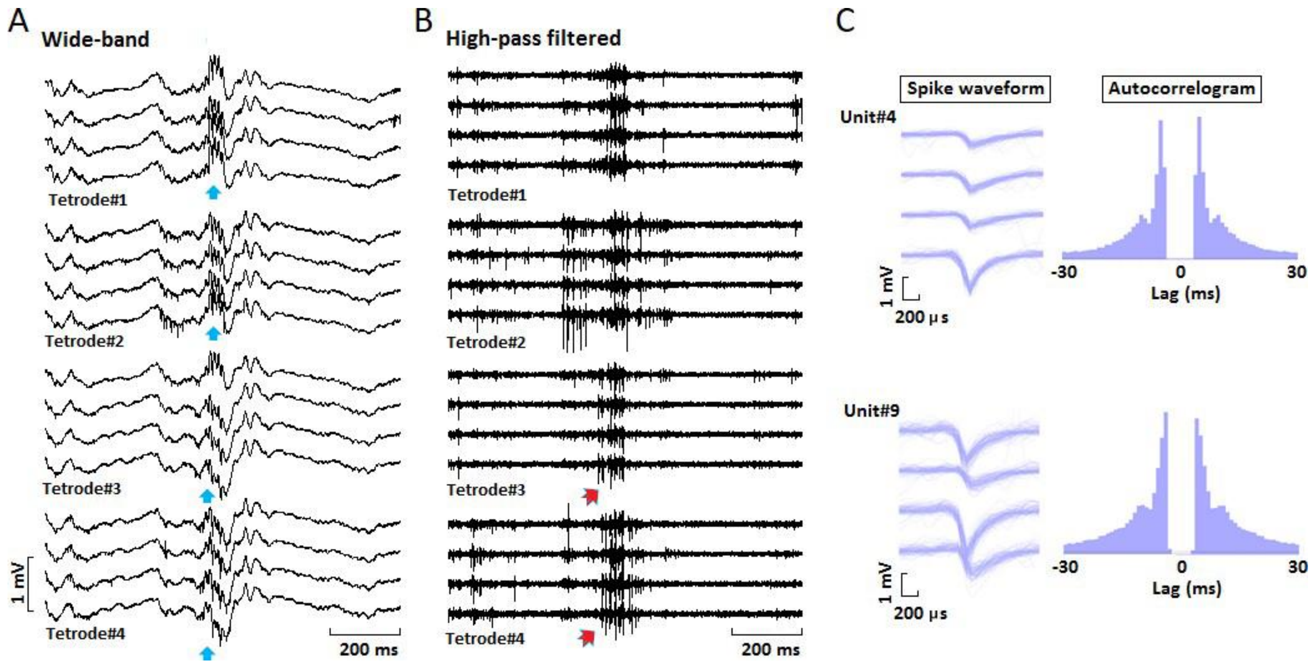

Fig. S2 *In vivo* identification of electrophysiological recording in dorsal hippocampus. **A** Example wide-band tetrode recording from the dorsal hippocampus (blue arrows, sharp wave-ripple (SWR) events). **B** High-pass (100–250 Hz) filtered traces from **A**, showing the activation of a cell assembly during SWR events (red arrows, hippocampal spikes associated with the occurrence of SWRs). **C** Spike waveforms and autocorrelograms for two representative units（unit #4 and #9）recorded from tetrodes #3 and #4.

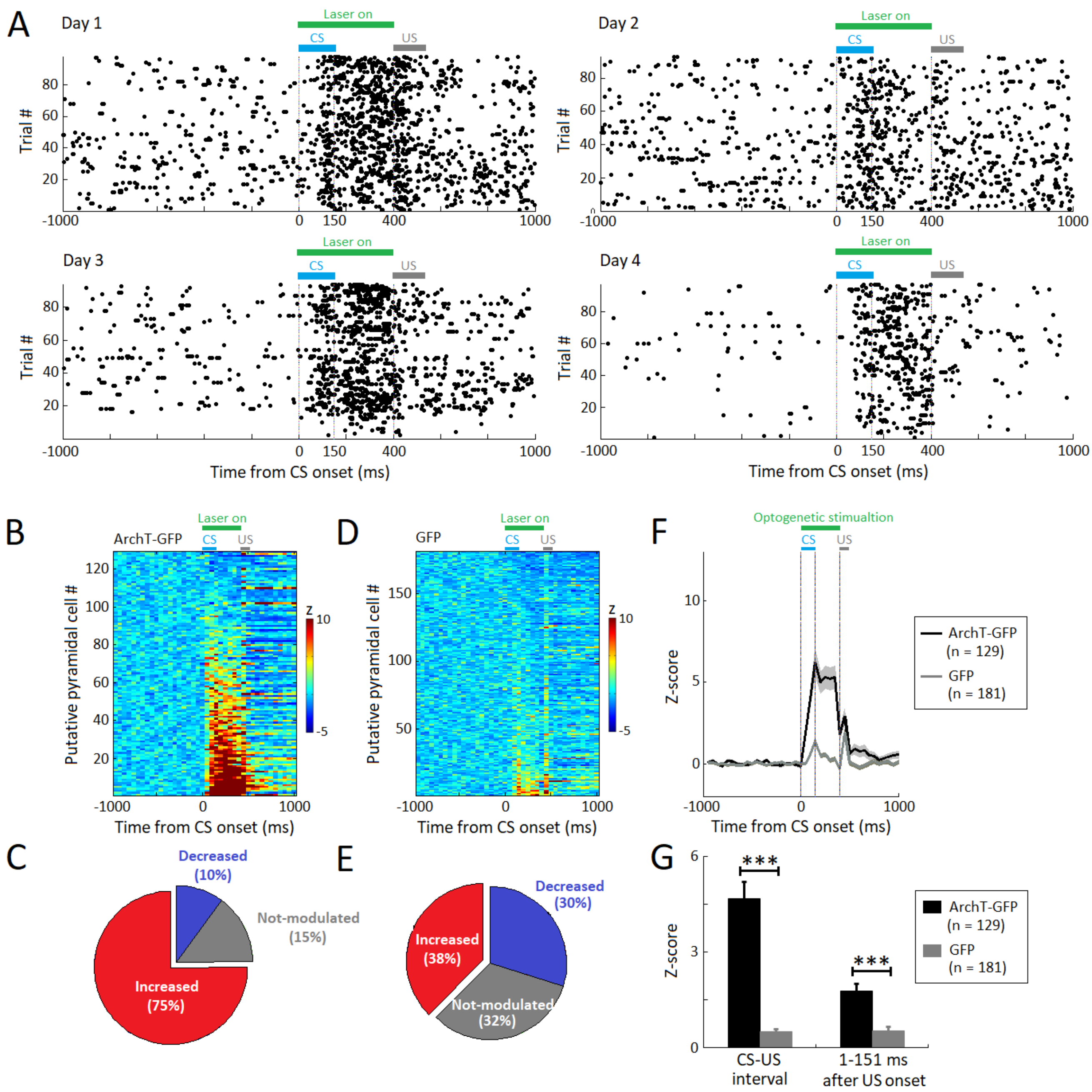

Fig. S3 Effect of optogenetic stimulation on the firing activity of putative pyramidal cells (Pyrs) during tEBC training. **A** Representative spike raster showing the effect of optogenetic stimulation on the firing activity of Pyrs during tEBC from day 1 to day 4. **B** Pseudocoloured, baseline normalized peri-CS histograms of all classified Pyrs (*n* = 129) during the acquisition of tEBC in mice expressing ArchT. Delivery of green laser light in the CS–US period was trigged by CS onsets. **C** Proportions of Pyrs showing increased (75%), decreased (10%), or minimal responses (15%) to optogenetic

stimulation in mice expressing ArchT. **D** Pseudocoloured, baseline normalized peri-CS histograms of all classified Pyrs (*n* = 181) during the acquisition of tEBC in mice expressing GFP. Delivery of green laser light in the CS–US period was trigged by CS onsets. **E** Proportions of Pyrs showing increased (38%), decreased (30%), or minimal responses (32%) to the CSs in mice expressing GFP. **F** Average Z scores of Pyr firing activity in mice expressing ArchT and GFP. **G** Left, Pyr firing activity during the CS-US period in mice expressing ArchT is greater than that in mice expressing GFP (ArchT-GFP: *n* = 129 units, GFP: *n* = 181 units, $t_{308} = 9.5435$, $P = 4.4413 \times 10^{-19}$, independent *t* test); right: Pyr firing activity during the 1–150 ms period after the US onset in mice expressing ArchT is greater than that in mice expressing GFP ($t_{308} = 5.2654$, $P = 2.6296 \times 10^{-7}$, independent *t* test). Therefore, the change of US-evoked activity is unlikely to be related to the CR impairment after optogenetic suppression. Data are expressed as the mean ± SEM (***P <0.001).
